# An Observational Study of Social and Emotional Support in Smoking Cessation Twitter Accounts: Content Analysis of Tweets

**DOI:** 10.2196/jmir.3768

**Published:** 2015-01-14

**Authors:** Mary Rocheleau, Rajani Shankar Sadasivam, Kate Baquis, Hannah Stahl, Rebecca L Kinney, Sherry L Pagoto, Thomas K Houston

**Affiliations:** ^1^Division of Health Informatics and Implementation ScienceQuantitative Health SciencesUniversity of Massachusetts Medical SchoolWorcester, MAUnited States; ^2^VA eHealth QUERI and CHOIRBedford, MAUnited States; ^3^Division of Preventive and Behavioral MedicineDepartment of MedicineUniversity of Massachusetts Medical SchoolWorcester, MAUnited States

**Keywords:** smoking cessation, Twitter, Internet, social network

## Abstract

**Background:**

Smoking continues to be the number one preventable cause of premature death in the United States. While evidence for the effectiveness of smoking cessation interventions has increased rapidly, questions remain on how to effectively disseminate these findings. Twitter, the second largest online social network, provides a natural way of disseminating information. Health communicators can use Twitter to inform smokers, provide social support, and attract them to other interventions. A key challenge for health researchers is how to frame their communications to maximize the engagement of smokers.

**Objective:**

Our aim was to examine current Twitter activity for smoking cessation.

**Methods:**

Active smoking cessation related Twitter accounts (N=18) were identified. Their 50 most recent tweets were content coded using a schema adapted from the Roter Interaction Analysis System (RIAS), a theory-based, validated coding method. Using negative binomial regression, the association of number of followers and frequency of individual tweet content at baseline was assessed. The difference in followership at 6 months (compared to baseline) to the frequency of tweet content was compared using linear regression. Both analyses were adjusted by account type (organizational or not organizational).

**Results:**

The 18 accounts had 60,609 followers at baseline and 68,167 at 6 months. A total of 24% of tweets were socioemotional support (mean 11.8, SD 9.8), 14% (mean 7, SD 8.4) were encouraging/engagement, and 62% (mean 31.2, SD 15.2) were informational. At baseline, higher frequency of socioemotional support and encouraging/engaging tweets was significantly associated with higher number of followers (socioemotional: incident rate ratio [IRR] 1.09, 95% CI 1.02-1.20; encouraging/engaging: IRR 1.06, 95% CI 1.00-1.12). Conversely, higher frequency of informational tweets was significantly associated with lower number of followers (IRR 0.95, 95% CI 0.92-0.98). At 6 months, for every increase by 1 in socioemotional tweets, the change in followership significantly increased by 43.94 (*P*=.027); the association was slightly attenuated after adjusting by account type and was not significant (*P*=.064).

**Conclusions:**

Smoking cessation activity does exist on Twitter. Preliminary findings suggest that certain content strategies can be used to encourage followership, and this needs to be further investigated.

## Introduction

While effectiveness evidence for smoking cessation interventions has increased rapidly [1-3], questions remain on how to effectively disseminate these findings [4]. The World Health Organization estimates that smoking causes the death of nearly 6 million people each year. Unless methods of reaching and engaging smokers are improved, the annual death toll could rise to more than 8 million by 2030 [5].

The potential of online social networks to disseminate health information has been recognized [6]. An estimated 73% of online adults in September 2013 used social networking sites. Of these, one in five adults went online to find others who might have health concerns similar to their own [7]. Twitter, in particular, provides a natural way of disseminating information. Created in 2006, Twitter is a live stream of news, opinions, and conversations [8]. Twitter allows users to communicate information through short messages called “tweets” consisting of a maximum of 140 characters. For many users, it has become their first source of information [8]. Health communicators can use Twitter to inform smokers, provide social support, and attract them to other interventions [9,10]. A key challenge for health researchers is how to frame their communications to maximize the engagement of smokers.

This study examined activities of Twitter accounts promoting smoking cessation. A content review was conducted of the tweets of these accounts and assessed the association between the tweet content and followership. We used a theoretically driven coding scheme—Roter Interaction Analysis System (RIAS)—which has been designed for biomedical and psychosocial content and is associated with important patient and provider outcomes [11]. Understanding this association may help in designing effective future interventions on Twitter.

## Methods

### Study Design

A retrospective examination of a cohort of active Twitter accounts promoting smoking cessation was conducted. This study was reviewed and determined to be non-human subjects research by the University of Massachusetts Medical School Institutional Review Board.

### Setting and Sample

A search for smoking cessation-related accounts was conducted on Twitter using the terms “quit smoking” and “smoking cessation”. Only accounts in English were considered for the sample. An inventory cohort of 130 smoking cessation Twitter accounts was identified. The date that the account was activated was determined by using the “how long have you been tweeting” Web service, which provides information about how long a Twitter account has been active [12]. From these data, the number of active days of the account was determined, along with the average number of tweets per day produced by each account. Accounts that averaged less than 1 tweet per day over the duration of the account’s life were eliminated. Accounts that did not tweet in the 24 hours prior to the time at which the inventory was taken were also eliminated. Based on these criteria, 18 accounts were included in the study (see Table 1).

### Content Coding of Tweets

There are several ways of coding communication, including using constructs from behavioral theory to guide the coding process. Behavior change theories frequently used in cancer prevention include Social Cognitive Theory, the Transtheoretical Model, and Theory of Reasoned Action [13]. Communication can also be coded based on clinical practice guidelines. Prochaska et al coded tweets based on clinical practice guidelines for treating tobacco dependence [14]. They can also be coded using a more inductive coding approach, by identifying and tagging content that may be pertinent to a specific user, including health and lifestyle status, health issues, and treatment options. In addition to coding for content, coding can be used to represent the structural aspects of the communication. Health messages are often evaluated on criteria studied in the field of health communication [15], including the types of appeals used in persuasive communication and their tone. Communication can also be coded in terms of complexity, including word count and literacy level.

We used a coding scale based on the RIAS motivational coding scheme. RIAS is a validated method of coding health communication and is associated with important patient and provider outcomes [11]. RIAS was designed to code communications and not to create effective messages. RIAS derives many concepts from social exchange theories related to interpersonal influence, problem solving, and reciprocity. RIAS provides mutually exclusive and exhaustive categories to code both the socioemotional, as well as the task-focused elements of communication. RIAS is proven to be practical, functional, flexible, and methodologically sound with high levels of reliability and predictive validity to a variety of outcome measures [11]. RIAS studies have demonstrated high levels of predictive and concurrent validity [16].

A subset of the RIAS codes was selected based on their applicability to the short message style of tweets. Seven mutually exclusive categories were used to code all tweets (see Table 2). Each tweet was independently coded by 2 coders. These coders were trained by authors (Pagoto and Houston) with prior experience in coding health communication. Initially, 84% agreement was achieved across both coders. All coding disagreements were resolved through a group review to achieve 100% agreement.

Tweets were categorized into three groups: (1) socioemotional support tweets, which included any tweet that involved personal remarks and reassuring statements, (2) encouraging/engaging tweets, which included the tweets categorized as gives orientation or suggestions, and ask open-ended questions, and (3) informational tweets, which promoted a product or event, as well as unrelated tweets that were not relevant to smoking.

**Table 1:** Account tweets, followers, and classification.

Organization	Twitter handle	Description	Active days	# tweets	Baseline followers	Followers at 6 months	Change in followers
Yes	@NICORETTE	Tweets from Nicorette	961	6336	15645	15568	-77
Yes	@FDATOBACCO	News updates from FDA Center for Tobacco Products	759	1285	10455	12779	2324
Yes	@SMOKEFREEWOMEN	Tweets from National Cancer Institute	1050	2866	7807	9928	2121
Yes	@QUITTEA	Tweets from the Quit Tea company	959	2324	6891	8509	1618
No	@how2quitsmoking	Tweets from successful quitter of 2 years	1200	18596	3544	3912	368
Yes	@QUITSMOKING	Tweets from Everyday Health, an online health information company (http://www.everydayhealth.com)	406	3851	3156	3021	-135
No	@smokefreelife	Tweets from an individual with the explanation: exploring the possibilities at the intersection of digital media & public health. Motto: Don't give up! Tweets/thinking my own	953	8926	3014	3213	199
Yes	@TRUTHORANGE	Tweets from truth.com, an organization against the tobacco industry	1184	1602	1986	3348	1362
Yes	@SMOKEFREEINDY	Tweets from Smoke Free Indy, a coalition of state, local public health, and community organizations dedicated to reducing secondhand smoke, tobacco usage, and tobacco initiation through education, prevention, and advocacy	1259	2127	1792	1936	144
No	@altersmoking	Smokers of 10 years trying to quit smoking for a year	847	2738	1733	1844	111
Yes	@QUITFULLSTOP_UK	Tweets from quitfullstop.co.uk, a Web-based smoking cessation site	217	507	1209	934	-275
Yes	@QUITSMOKINGSOON	Tweets from http://quitsmokingonlineblog.blogspot.com/, a resource for quit smoking related articles	883	1156	753	859	106
No	@quitsmoking6	Tweets providing useful tips and advice to help users quit smoking	424	3724	589	612	23
No	@quit_smokin_now	Tweets about best ways to quit smoking	863	2392	586	586	0
Yes	@SMOKEFREEPCT	Tweets from the NHS West Kent SmokeFree Service, a specialist team helping local people to quit smoking for free http://www.smokefreewestkent.co.uk	706	729	453	0	-453
No	@quitsmokingform	Tweets providing information to help users to stop smoking	621	2161	342	354	12
Yes	@QUIT_SMOKING_OW	Organization sharing smoking cessation resources shared by health experts, advocates, and organizations into wisdom cards	1359	5174	340	403	63
Yes	@MNT_SMOKING	The latest smoking & quit smoking news published daily; articles from research centers, universities, and prestigious journals; http://www.medicalnewstoday.com/sections/smoking/	888	955	314	361	47

**Table 2:** Coding scheme of tweet content.

Code grouping	Code and definition	Codes/Tweet (N=900), n (%)	Example	
Socioemotional support	Personal remarks, social conversation: Success stories, thanking other users for following	125 (13.9)	@StopSmokingCIOS Thanks for the link! Yes, we've seen them. Very thought provoking. 10 Years - Full Circle: At 10 years smoke-free, Michelle has plenty to say about how she quit, and the benefits...http://bit.ly/z8gIkC	
	Reassures, encourages, or shows optimism: Any tweet related to motivation, inspirational quotes	87 (9.7)	Finally, it's Saturday! Wishing you all a healthy and happy weekend. Make sure to pack it full of motivational activities.	
Encouraging/ engaging	Gives orientation, instructions, suggestions: How to, any tips related to cravings, smoking cessation, and long-term success with quitting	91 (10.1)	#Tip Know your triggers. Create a plan for each. Exmpl: Smoke after meals-->Wash dishes, brush teeth, take a short walk to break routine.	
	Asks open-ended questions: Any tweet that prompts a response or encourages interactions between users	35 (3.9)	How many days into quitting are you? Tweet at us, and we'll share for inspiration! #ThisIsYOURYear	
Informational	Promotion of a product or event: Any tweet that mentions or endorses a product, or encourages attendance of an event on a specific date	122 (13.6)	It's a new day. A new week. A new you. Try to quit smoking today with @quittea http://ow.ly/cgqTt http://ow.ly/i/LZyy Retweeted by Quit Tea	
	Unrelated comments: Does not explicitly mention smoking or smoking cessation methods	52 (5.8)	Heidi Klum and Seal separate: when's the downward spiral of celeb divorce going to end? http://trib.al/jUQ9Sz	
	Gives information on a medical condition: Specific mention of a disease or condition related to smoking (lung cancer, respiratory problems)	48 (5.3)	NYT: Smoker presents w/ coughing fits & holes in bones: pulmonary Langerhans cell histiocytosis #PLCH. http://ow.ly/ciyFH	
	Gives information on lifestyle: Day-to-day effects of quitting smoking including dietary changes, exercise suggestions, and smoking alternatives	83 (9.2)	Once you quit: Your bad breath is gone. The stains on your teeth, fingers, and fingernails fade. You have more overall energy to enjoy life.	
	Gives information on psychosocial: Related to changing behavior as a result of social interactions, environment, and individual thoughts	38 (4.2)	Can Facebook Make You Quit Smoking The Daily Beast http://bit.ly/NpqaWQ	
	Gives information on news: New developments related to quit smoking technology, recently published journal articles	174 (19.3)	ABC Nightline News: Is #BigTobacco profiting from kids? #Video. http://ow.ly/cj1lP	
	Gives information on other: Contains content unrelated to a medical condition, lifestyle habit, psychosocial factor, or news	45 (5.0)	Indonesia Zoo Helping Orangutan Quit Smoking After 10 Years (Video) http://bit.ly/NIBp8V	

### Data Collection

We considered several methods for selecting tweets. We needed a sufficient number of tweets to achieve a stable within account estimates. RIAS has been found to be conservative resource making it possible to conduct research with smaller sample sizes [16]. In prior studies using RIAS, number of within cluster measures ranged from 6-20 [17-22].

Thus we chose 50 tweets. We considered a random sample, but because number of tweets varied by account and by time, we chose the 50 most recent tweets to reflect current account activity. An inventory of the 50 most recent tweets was manually collected for each of the selected 18 accounts on July 18, 2012. The median number of days for the 50 tweets was 27 (intraquartile range 10.75-48). From the account’s homepage, we collected the number of followers that each account had at baseline and at 6 months. The type of account was also identified: Organization or Not Organization. Accounts that specifically stated that they represent or are associated with an organization, product, or initiative were classified as Organization accounts. Accounts owned by an individual tweeting about their experience with smoking cessation, or accounts that did not specifically relate to an organization, product or initiative, were classified as Not Organization accounts.

### Data Analysis

#### Cross-Sectional Association Between Number of Followers and Frequency of Tweet Content

A cross-sectional comparison of the association of number of followers (dependent variable) and frequency of individual tweet content (independent variable) at baseline was performed. We used a negative binomial regression model due to over-dispersion of the variance of the distribution of the dependent variable.

#### Longitudinal Analysis of Change in Followers and Frequency of Tweet Content

The change in followership was compared to the frequency of tweet content using linear regression. We calculated the change in followership as the difference in the number of followers of an account at follow-up (at 6 months) compared to baseline.

One challenge in using the absolute difference in followers is that this crude measure does not account for the size of followership at baseline. Thus, a new measure was developed—followership ratio. The followership ratio was calculated as the observed change in followership for a specific account divided by the mean change in followership for all accounts (ie, actual/expected).

Analyses were adjusted for by account type, and all analyses were performed using Stata version 11.

## Results

### Account Characteristics

The 18 accounts had 60,609 followers at baseline; 68167 at 6 months. More than half (12/18, 67%) of the accounts were organizations. Six could not be clearly identified as organizations and may represent individual accounts. Mean number of days the accounts had been active was 863 (SD 306, range 217-1359). Over the duration of their existence, these 18 accounts sent a mean number of 3747.17 tweets (SD 4281, range 507-18596). At baseline, the accounts had a mean followership of 3367 (SD 4224, range 314-15645); at 6 months the followership changed to 3787 (SD 4692, range 0-15568). One organization account had closed at 6 months (Table 1).

### Content Coding of Tweets

As noted, the total number of tweets was 900. We found that 13.9% (125/900, mean 6.9, SD 8.5) of tweets were personal remarks or social conversation, 9.7% (87/900, mean 4.8, SD 5.0) reassured, encouraged, or showed optimism, 10.1% (91/900, mean 5.1, SD 5.6) gave orientation, instructions, or suggestion, and 13.6% (122/900, mean 6.8, SD 10.0) promoted a product or event. Very few of the tweets (5.8%, 52/900, mean 2.9, SD 4.2) were unrelated to smoking cessation (Table 2). In fact, 23.6% of tweets were socioemotional support (212/900, mean 11.8, SD 9.8), 14.0% (126/900, mean 7, SD 8.4) were encouraging/engagement, and 62.4% (562/900, mean 31.2, SD 15.2) were informational.

### Cross-Sectional Association Between Number of Followers and Frequency of Tweet Content

At baseline, after adjustment for account type, tweets with higher frequency of reassuring messages were significantly associated with higher number of followers (incident rate ratio [IRR] 1.14, 95% CI 1.03-1.26) (Table 3). Higher frequency of socioemotional support and encouraging/engaging tweets was also associated with higher number of followers (socioemotional: IRR 1.09, 95% CI 1.02-1.16; encouraging/engaging: IRR 1.06, 95% CI 1.00, 1.11). Higher frequency of informational tweets was significantly associated with lower number of followers (IRR 0.95, 95% CI 0.92-0.98).

**Table 3:** Association of number of followers and frequency of tweets.

	IRR (95% CI)	IRR (95% CI) after adjustment by account type
Personal	1.03 (0.97-1.09)	1.02 (0.96-1.08)
Reassure	1.15 (1.04-1.26)^b^	1.14 (1.03-1.26)^a^
Suggest	1.06 (0.96-1.18)	1.06 (0.97-1.17)
Question	1.08 (0.99-1.18)	1.08 (0.99-1.17)
Info	0.94 (0.92-0.97)^b^	0.95 (0.92-0.97)^b^
Product	0.98 (0.93-1.04)	0.99 (0.94-1.05)
Unrelated	0.97 (0.87-1.08)	1.01 (0.88-1.16)
Socioemotional	1.08 (1.03-1.14)^b^	1.09 (1.02-1.16)^b^
Encourage/Engage	1.06 (1.01-1.11)^a^	1.06 (1.00-1.11)^b^
Informational	0.95 (0.92-0.97)^b^	0.95 (0.92-0.98)^a^

^a^ *P*<.05.

^b^ *P*<.01.

### Longitudinal Analysis of Change in Followers and Frequency of Tweet Content

The longitudinal analysis was conducted first using change in followers—the difference in the number of followers at follow-up (compared to baseline). The median change in followers was 84.50 (interquartile range 19.25-616.50). For every increase by 1 in socioemotional tweets, the change in followers increased by (beta coefficient 43.94, *P*=.027); the association was slightly attenuated after adjusting by account type and was not significant (*P*=.064). For every increase by 1 in socioemotional tweets, the change in followers increased by (beta coefficient 43.94, *P*=.027); the association was slightly attenuated after adjusting by account type and was not significant (*P*=.064). For every increase by 1 encouraging/engaging and informational tweets, the change in followers decreased (encouraging/engaging tweets: beta coefficient -0.33, *P*=.99; informational tweets: beta coefficient -18.30, *P*=.175).

Additionally, we conducted a longitudinal analysis using the followership ratio calculated as the observed or the actual change in followership over the expected or the mean change in followership. The median followership ratio was 0.20 (interquartile range -0.04-1.50). For every increase by 1 in socioemotional tweets, the followership ratio increased by (beta coefficient 0.10, *P*=.027). The association was slightly attenuated after adjusting by account type and was not significant (*P*=.064). For every increase by 1 encouraging/engaging and informational tweets, the change in followers decreased (encouraging/engaging tweets: beta coefficient -0.0008, *P*=.99; informational tweets: beta coefficient -0.043, *P*=.175). After adjustment, this did not change.

##  Discussion

### Principal Findings

Numerous accounts exist that promote smoking cessation on Twitter. The accounts identified in this study had 60,609 followers in total. The content of the accounts was informational and also included socioemotional and encouraging/engaging tweets. Interestingly, socioemotional content was associated with increased number of followers at baseline and over 6 months, while accounts that tweeted mostly informational tweets about the harmful effects of smoking had fewer followers. Identifying strategies that increase engagement is an important social networking and public health question [4]. Future intervention research on Twitter should compare different content strategies on engagement and smoking cessation outcomes.

Twitter has been used to recruit subjects for health behavioral studies [23,24] and deliver health-related social support [25,26]. Other studies have reviewed Twitter account activity [10,27-30]. Prochaska et al coded the tweets based on clinical practice guidelines for tobacco treatment dependence. They classified the tweets into three major categories (personal communications to support cessation, postings via an automatic newsfeed, or links to commercial sites for purchase of cessation products) [10]. A significant correlation between total tweets and followers (Spearman rho=.57, *P*<.001), number of active days and followers (Spearman rho=.48, *P*<.001), and number of active days and total tweets per account (Spearman rho=.23, *P*=.005) was found. In addition to adapting a standard health communication coding schema for coding tweets, our analysis is unique in that we also longitudinally assessed the association between content and followership.

Furthermore, this study also has a methodological contribution. A new estimate (followership ratio) was developed to account for the size of population at baseline. Similar ratios such as the Standardized Mortality Rates are used outside the social networking research to account for a change in a factor of a subgroup with respect to the general population [31]. Although the results of this study using the new estimate were not different from the crude absolute measure that was also used (change in followership), other studies might have different results. Additional research is needed to further study and develop this new followership ratio estimate, which is an important area in social networking research [4].

### Limitations

One limitation of this study was the sample size. Only 18 relevant accounts were identified. Additionally, only 50 tweets were viewed in a snapshot of time per account, and these might not be representative of the account. The goal was to assess tweets at a particular instance in time and then to prospectively look at followership at 6 months. Thus, it may not represent everything that happened within the account. Additionally, to achieve a sufficient number of tweets to achieve a stable within-account estimate, we chose 50 of the most recent tweets, not a random sample of tweets. Furthermore, this study did not assess whether these accounts had any impact on cessation efforts. It is also unknown if followers of these accounts are primarily smokers.

### Conclusions

Twitter has the potential to be a new channel for smoking cessation interventions. Although easily accessible, evidenced-based tools exist in smoking cessation, they are underused [32-35]. Current recruitment methods such as search engine advertisements are limited in that they require the user to initiate the contact and come to the intervention [32-35]. Delivering the intervention where smokers are already engaged could be a more effective engagement approach. This study further highlights the potential of Twitter as a smoking cessation resource and indicates certain content strategies that can be used to encourage followership. Further research is needed to assess whether smokers engaged on Twitter can also be encouraged to use additional cessation resources such as a Web-assisted tobacco intervention.

## Abbreviations

IRR: incident rate ratio
RIAS: Roter Interaction Analysis System

## References

[1] Myung SK, McDonnell DD, Kazinets G, Seo HG, Moskowitz JM (2009). Effects of Web- and computer-based smoking cessation programs: meta-analysis of randomized controlled trials. Arch Intern Med.

[2] Shahab L, McEwen A (2009). Online support for smoking cessation: a systematic review of the literature. Addiction.

[3] Civljak M, Sheikh A, Stead LF, Car J (2010). Internet-based interventions for smoking cessation. Cochrane Database Syst Rev.

[4] Cobb NK, Graham AL, Byron MJ, Niaura RS, Abrams DB, Workshop Participants (2011). Online social networks and smoking cessation: a scientific research agenda. J Med Internet Res.

[5] World Health Organization (2014). WHO: Tobacco.

[6] Pew Internet Research Project (2013). 72% of Online Adults are Social Networking Site Users.

[7] Fox S (2011). Pew Internet Research Project.

[8] Akshay J, Xiaodan S, Tim F, Belle T (2007). Why we twitter: understanding microblogging usage and communities.

[9] Cook S, Dean E, Richards AK, Haque SN (2012). What’s Happening?" Twitter for Diary Studies.

[10] Prochaska JJ, Pechmann C, Kim R, Leonhardt JM (2012). Twitter=quitter? An analysis of Twitter quit smoking social networks. Tob Control.

[11] Roter D, Larson S (2002). The Roter interaction analysis system (RIAS): utility and flexibility for analysis of medical interactions. Patient Educ Couns.

[12] How long have you been tweeting?.

[13] US Department of Health and Human Services Making health communication programs work.

[14] Tobacco Use and Dependence Guideline Panel (2008). Treating Tobacco Use and Dependence: 2008 Update.

[15] Maibach E, Parrott R (1995). Designing health messages: approaches from communication theory and public health practice.

[16] Roter D, Larson S (2002). The Roter interaction analysis system (RIAS): utility and flexibility for analysis of medical interactions. Patient Educ Couns.

[17] Beach MC, Roter DL, Wang NY, Duggan PS, Cooper LA (2006). Are physicians' attitudes of respect accurately perceived by patients and associated with more positive communication behaviors?. Patient Educ Couns.

[18] Bensing J, Verheul W, van Dulmen A (2008). Patient anxiety in the medical encounter. Health Education.

[19] Mjaaland TA, Finset A (2009). Communication skills training for general practitioners to promote patient coping: the GRIP approach. Patient Educ Couns.

[20] Greer RC, Cooper LA, Crews DC, Powe NR, Boulware LE (2011). Quality of patient-physician discussions about CKD in primary care: a cross-sectional study. Am J Kidney Dis.

[21] Albada A, Ausems MG, van Dulmen S (2014). Counselee participation in follow-up breast cancer genetic counselling visits and associations with achievement of the preferred role, cognitive outcomes, risk perception alignment and perceived personal control. Soc Sci Med.

[22] Daaleman TP, Shea CM, Halladay J, Reed D (2014). A method to determine the impact of patient-centered care interventions in primary care. Patient Educ Couns.

[23] Leonard A, Hutchesson M, Patterson A, Chalmers K, Collins C (2014). Recruitment and retention of young women into nutrition research studies: practical considerations. Trials.

[24] O'Connor A, Jackson L, Goldsmith L, Skirton H (2014). Can I get a retweet please? Health research recruitment and the Twittersphere. J Adv Nurs.

[25] Pagoto SL, Schneider KL, Oleski J, Smith B, Bauman M (2014). The adoption and spread of a core-strengthening exercise through an online social network. J Phys Act Health.

[26] Turner-McGrievy GM, Tate DF (2013). Weight loss social support in 140 characters or less: use of an online social network in a remotely delivered weight loss intervention. Transl Behav Med.

[27] Heaivilin N, Gerbert B, Page JE, Gibbs JL (2011). Public health surveillance of dental pain via Twitter. J Dent Res.

[28] De la Torre-Díez I, Díaz-Pernas FJ, Antón-Rodríguez M (2012). A content analysis of chronic diseases social groups on Facebook and Twitter. Telemed J E Health.

[29] Donelle L, Booth RG (2012). Health tweets: an exploration of health promotion on twitter. Online J Issues Nurs.

[30] Thackeray R, Burton SH, Giraud-Carrier C, Rollins S, Draper CR (2013). Using Twitter for breast cancer prevention: an analysis of breast cancer awareness month. BMC Cancer.

[31] Wolfe RA (1994). The standardized mortality ratio revisited: improvements, innovations, and limitations. Am J Kidney Dis.

[32] Koo M, Skinner H (2005). Challenges of internet recruitment: a case study with disappointing results. J Med Internet Res.

[33] Graham AL, Milner P, Saul JE, Pfaff L (2008). Online advertising as a public health and recruitment tool: comparison of different media campaigns to increase demand for smoking cessation interventions. J Med Internet Res.

[34] Murray E, Khadjesari Z, White IR, Kalaitzaki E, Godfrey C, McCambridge J, Thompson SG, Wallace P (2009). Methodological challenges in online trials. J Med Internet Res.

[35] Jones RB, O'Connor A, Brelsford J, Parsons N, Skirton H (2012). Costs and difficulties of recruiting patients to provide e-health support: pilot study in one primary care trust. BMC Med Inform Decis Mak.

